# Familiarity with social sounds alters c-Fos expression in auditory cortex and interacts with estradiol in locus coeruleus

**DOI:** 10.1016/j.heares.2018.06.020

**Published:** 2018-06-28

**Authors:** Amielle Moreno, Ankita Gumaste, Geoff K. Adams, Kelly K. Chong, Michael Nguyen, Kathryn N. Shepard, Robert C. Liu

**Affiliations:** a Neuroscience Graduate Program, Emory University, 1462 Clifton Road, Atlanta, GA, 30322, USA; b Department of Biology, Emory University, 1510 Clifton Road, Atlanta, GA, 30322, USA; c Neuroscience and Behavior Biology Program, Emory University, 1462 Clifton Road, Atlanta, GA, 30322, USA; d Biomedical Engineering Graduate Program, Georgia Institute of Technology, North Ave NW, Atlanta, GA, 30332, USA; e Center for Translational Social Neuroscience, Emory University, Atlanta, GA, 30322, USA

**Keywords:** Estrogen, Social learning, Stability, Novelty, Plasticity, Experience dependent, Maternal behavior, Hormone, Ultrasonic, Vocalization, Sensory

## Abstract

When a social sound category initially gains behavioral significance to an animal, plasticity events presumably enhance the ability to recognize that sound category in the future. In the context of learning natural social stimuli, neuromodulators such as norepinephrine and estrogen have been associated with experience-dependent plasticity and processing of newly salient social cues, yet continued plasticity once stimuli are familiar could disrupt the stability of sensorineural representations. Here we employed a maternal mouse model of natural sensory cortical plasticity for infant vocalizations to ask whether the engagement of the noradrenergic locus coeruleus (LC) by the playback of pup-calls is affected by either prior experience with the sounds or estrogen availability, using a well-studied cellular activity and plasticity marker, the immediate early gene c-Fos. We counted call-induced c-Fos immunoreactive (cFos-IR) cells in both LC and physiologically validated fields within the auditory cortex (AC) of estradiol or blank-implanted virgin female mice with either 0 or 5-days prior experience caring for vocalizing pups. Estradiol and pup experience interacted both in the induction of c-Fos-IR in the LC, as well as in behavioral measures of locomotion during playback, consistent with the neuromodulatory center’s activity being an online reflection of both hormonal and experience-dependent influences on arousal. Throughout core AC, as well as in a high frequency sub-region of AC and in secondary AC, a main effect of pup experience was to reduce call-induced c-Fos-IR, irrespective of estradiol availability. This is consistent with the hypothesis that sound familiarity leads to less c-Fos-mediated plasticity, and less disrupted sensory representations of a meaningful call category. Taken together, our data support the view that any coupling between these sensory and neuromodulatory areas is situationally dependent, and their engagement depends differentially on both internal state factors like hormones and external state factors like prior experience.

## 1. Introduction

Conspecific vocalizations are one of the most ethologically important auditory cues in an animal’s environment. While evolutionary forces likely predispose sensory systems to respond innately to these signals (Garcia-Lazaro et al., 2015), recent research suggests sensorineural plasticity also plays a role in enhancing such responses (Shepard et al., 2015b) as individuals gain familiarity with a vocal category through experience. Learning the relevance of a vocal category can improve future recognition and behavioral response (Poremba et al., 2013; Tsunada and Cohen, 2014). Plasticity events in auditory cortical and neuromodulatory areas are hypothesized to facilitate future processing to ensure robust behavioral response (Galindo-Leon et al., 2009; Ivanova et al., 2017). However, driving new plasticity for increasingly salient sounds must also be balanced against the need to maintain established sensorineural representations. How sensory plasticityrelated regions are engaged as a function of prior experience is still not fully understood.

Neuromodulators influence neural circuits that process ethologically relevant stimuli (Bargmann, 2012). One such neuromodulator is norepinephrine (NE), which is associated with behavioral arousal and facilitates sensory cortical plasticity (Berridge and Waterhouse, 2003; Edeline et al., 2011; Lynch et al., 2012; Ikeda et al., 2015; Shepard et al., 2015a; Martins and Froemke, 2015). NE also plays a role in mediating social memories (Griffin and Taylor, 1995; Thomas and Palmiter, 1997; Marino et al., 2005; Pissonnier et al., 1985), raising the likelihood that arousing social sounds could engage the noradrenergic system to drive sensory cortical plasticity and facilitate behavioral responses.

Estradiol (E2), a steroidal hormone, is also strongly implicated in social behavior and plasticity. E2 is associated with improved performance on memory tasks like social recognition (Phan et al., 2011) and processing behaviorally relevant conspecific vocalizations (Maney et al., 2008; Remage-Healey et al., 2008, 2010). In mice, estrogen receptors are found along the auditory pathway (Charitidi and Canlon, 2010), and E2 administration in pup-sensitized virgin females facilitates maternal approach behavior and recognition of model pup-calls (Koch and Ehret, 1989; Ehret and Haack, 1984), though whether E2 acts on neuromodulatory or auditory areas to exert this influence is still unclear.

One hypothesis is E2 influences sensory cortex plasticity through actions on neuromodulatory systems (Miranda and Liu, 2009). Research in songbirds suggests noradrenergic neurons target sites with E2 receptors and express estrogen receptor mRNA (Heritage et al., 1980; Shughrue et al., 1997). E2 administration modulates the concentration of NE in the zebra finch song system (Wade et al., 2013) and the ewe ovine preoptic area (Goodman et al., 1995), as well as the mRNA of the enzyme that creates NE in the rat locus coeruleus (LC, (Serova et al., 2011)). Further, E2 plays a role in structural changes in catecholaminergic fibers within the songbird auditory forebrain (Matragrano et al., 2011; Appeltants et al., 2004). Hence, E2 could indirectly modulate the processing of salient auditory stimuli by influencing noradrenergic neurons in the LC.

These documented roles for both NE and E2 in social sensory processing and plasticity, as well as their relationship to one another, led us here to ask how these neurochemicals affect neural responses during playback of novel or familiar social auditory cues, using a maternal mouse model of social auditory learning. A growing body of literature suggests the detection of infant cues by mothers may be facilitated by maternal sensory plasticity in association with maternal hormones, including oxytocin and potentially E2 (Banerjee and Liu, 2013; Marlin et al., 2015; Lin et al., 2013). There is improved detection, discrimination and categorization of infant pup vocalizations in the maternal auditory cortex (AC), persisting after maternal experience (Lin et al., 2013; Liu and Schreiner, 2007; Cohen et al., 2011; Galindo-Leon et al., 2009; Shepard et al., 2016). Here we asked whether E2 availability, manipulated systemically with subcutaneous implants, modulates how the noradrenergic LC responds when virgin ovariectomized females hear pup ultrasonic isolation vocalizations, either for the first time as adults (novel) or after social experience raising pups so that the call category is familiar. We measured the expression of the plasticity-associated immediate early gene (IEG) *c-fos* (Dragunow and Faull, 1989; Sagar et al., 1988; Montag-Sallaz et al., 1999) in the LC as well as the AC (de Hoz et al., 2017).

Our findings suggest E2 and familiarity with the social vocalizations affect c-Fos immunoresponsivity (c-Fos-IR) in neuromodulatory and auditory cortical processing regions in distinct ways. E2 and social experience interacted to drive both LC c-Fos-IR and locomotion measures, consistent with this neuromodulatory center playing a role in immediate behavioral responses to arousing stimuli. Meanwhile, the AC showed generally decreased c-Fos-IR in animals familiar with pup-calls, irrespective of E2 availability, consistent with a sensory cortical role in maintaining a more stable representation of stimuli that have gained behavioral relevance. Thus, after social sounds have become familiar, the genomic responses in LC and AC reflect complementary roles these areas play, respectively, in these auditory cues’ salience versus memory.

## 2. Materials and methods

### 2.1. Animals

The Emory University Institutional Animal Care and Use Committee approved all procedures involved in this study. Experiments were performed on adult virgin female *CBA/CaJ* mice. Animals were weaned at 21 days, placed in single-sex ALPHA-dri bedded housing with two to five animals per cage under a reverse-light cycle (14 h of light/10 h of dark), and had access to food and water *ad libitum*. Females were between 12 and 14 weeks of age at the time of pup-call sound exposure.

### 2.2. Hormonal manipulation

**Fig 1.**
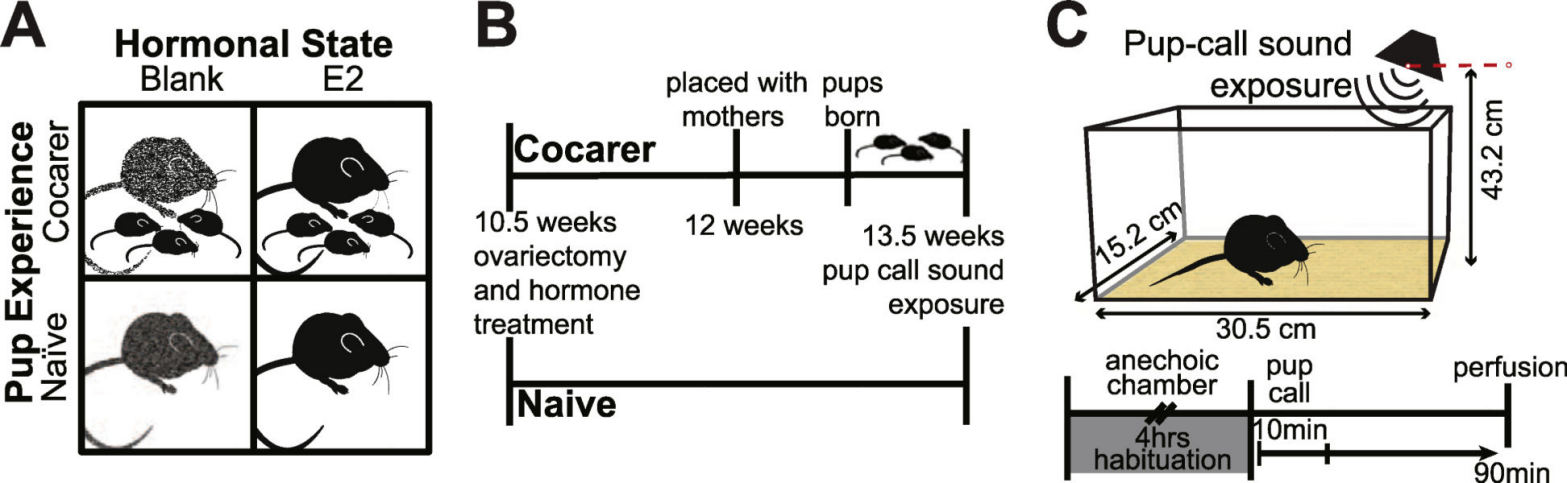
Animal experimental conditions and timelines. **(A)** Schematic of the four experimental groups used. Columns represent hormonal treatment (speckled = blank implant (B), solid = estradiol (E2) implant). Rows represent pup experience (top row = cocarers, bottom row = naïve) **(B)** Timeline of experimental design **(C)** Sound exposure cage set up and timeline.

Animals in our 2 × 2 design (Fig. 1A) were ovariectomized and randomly assigned to be implanted with a subcutaneous capsule (2 mm Silastic tubing, sealed with silicone aquarium sealant) containing either estradiol benzoate (E2) dissolved in sesame oil (50 μl at 3 mg/ml; n = 42), or a blank (B; n = 43) control containing only sesame oil. Prior to implantation, all capsules were soaked in 0.9% saline solution and sterilized using hydrogen peroxide gas sterilization for 29 minutes (min).

On the day of experimentation, animals were an average of 19 ± 4 (mean ± standard devidation) days after hormone (or vehicle) implant surgery. The E2 implant concentration was chosen because two weeks after implantation, animals treated with this dose have plasma E2 levels comparable to mothers immediately before parturition (Barkley et al.,1979; McCormack and Greenwald, 1974; Miranda et al., 2014). Animals were housed individually after surgery for recovery. In seven additional animals, ovariectomy and implantation of estradiol (n = 3) and blank (n = 4) capsules was performed and then the serum levels of estradiol in these animals tested 21 days later. Results confirmed that estradiol implanted animals had a significantly higher concentration of serum estradiol than the blank implanted animals (*p* < 0.01).

### 2.3. Pup experience manipulation

Blank and E2-implanted animals were assigned to one of two groups with different levels of pup experience: naïve (N) or cocarer (CC). Naïve animals were singly housed and not given any adult experience with pups. Cocarer animals were placed at around 12 weeks of age, after ovariectomy/implantation surgery and recovery, in a cage with a pregnant female littermate shortly before birth (Fig. 1B). Cocarers spent 5e6 days caring for pups with the mother before being individually housed ahead of sound exposure the next day (Ehret et al., 1987).

### 2.4. Stimulus presentation

In mice, pups produce characteristic isolation calls when removed from the nest (Liu et al., 2003), which in turn elicit a maternal response from dams to find the vocalizing pups (Haack et al., 1983). On the day following separation into individual housing, each cocarer or naïve mouse in its home cage was placed in a sound-attenuating chamber (IAC Acoustics) equipped with a speaker (Fig. 1C). All experimentation took place during the dark phase of the light cycle under red light. After an acclimation period of 4 hours (h), we played a 10-min recording of natural ultrasonic *CBA/CaJ* pup isolation calls (n = 65 total animals, blank/naïve = 17, estradiol/naïve = 15, blank/cocarer = 16, estradiol/cocarer = 18) or a 10-min background noise recording (blank/naïve = 5, estradiol/ naïve = 4, blank/cocarer = 5, estradiol/cocarer = 4). The pup isolation call recording consisted of concatenated one-min bouts extracted from 10 different pups (Liu et al., 2003), sampled at 223 kS/s, an average of 55 dB SPL with some calls reaching 95 dB SPL and high-pass filtered above 25 kHz to attenuate low frequency noise. The background noise stimulus consisted of 10-min-long segments from the pup isolation recordings, an average of 42 dB SPL that were also high-pass filtered above 25 kHz and clipped to exclude any pup vocalizations. An ultrasonic bat detector (Ultrasound Advice, MINI-3) was used to ensure the successful playback of the pup isolation calls. All animals remained in their respective sound-attenuating chambers for an additional 80 min of silence following the 10-min stimulus to allow for immediate early gene (IEG) induction and expression (Chaudhuri et al., 2000). Animals were then euthanized using carbon dioxide and transcardially perfused with KPBS and 4% paraformaldehyde over the course of 10 min.

As described in the Results, c-Fos-IR in response to the background noise was assayed for the LC, but not the AC. Research on immediate early genes from our laboratory showed that our type of background sound, while novel, does not elicit differences across pup experienced and inexperienced groups (Ivanova et al., 2017). Hence, here we focused in AC on comparing hormone and experience effects on the response to the *same* social sound stimulus.

### 2.5. Immunohistochemistry for tyrosine hydroxylase and c-Fos

Brains were removed from the skulls and post-fixed overnight in 4% paraformaldehyde, cryoprotected in 30% sucrose until no longer floating, and stored at 4C°. Brains were coronally sectioned at 50 microns (μm), and sections containing the LC or AC were stored in cryoprotectant at −20C°. Double-label immunohistochemistry using both c-Fos antibody (polyclonal *c-Fos* antibody raised in rabbit; Santa Cruz Biotechnology, Santa Cruz, CA, USA; cat. No. sc-52) and tyrosine hydroxylase (TH) antibody (Immunostar, Hudson, WI, USA; cat. No. 22941) was conducted on every other LC section. Single labeling immunohistochemistry using c-Fos antibody was conducted on every other AC section. The double-label procedure was carried out as follows: free-floating sections were washed in PBS, incubated for 15 min in 0.1% sodium borohydride, washed again in PBS, and rinsed for 30 min in 3% H_2_O_2_. Sections were then washed in PBST (PBS with 0.3% Triton-X 100) and blocked using 20% normal goat serum (NGS) in PBST for 1 h. Sections were then transferred to c-Fos primary antibody at a dilution of 1:2000 in PBSTN (0.3% triton, 2% NGS) and stored at 4C° for 2 days with gentle agitation. After 2 days of incubation with c-Fos antibody, sections were rinsed in PBSTN and incubated for 1 h in biotinylated goat anti-rabbit IgG secondary antibody (Vector Laboratories, Burlingame, CA, USA; cat. No.BA-1000) diluted to 1:250 in PBSTN. Sections were then washed in PBST and incubated in an ABC solution diluted to 1:200 (Vector Laboratories, Burlingame, CA, USA; cat. No. PK-6100) for 1 h. Sections were washed in PBS, rinsed in acetate buffer, and protein expression was visualized using nickel-enhanced diaminobenzidine (niDAB). Multiple acetate buffer washes were complete before PB and then rinsing in 3% H_2_O_2_ for 20 min. Sections were then rinsed in PBTN and transferred to TH antibody at a dilution of 1:1000 in PBTN and stored at 4C° for 2 days. After 2 days of incubation with TH antibody, sections were rinsed in PBTN and incubated for 1 h in biotinylated goat antimouse IgG secondary antibody (Vector Laboratories, Burlingame, CA, USA; cat. NO. BA-9200) diluted to 1:250 in PBTN. Sections were then washed in PBT and incubated in an ABC solution for 1 h. Following ABC incubation, sections were washed in PB and protein expression was visualized using diaminobenzidine. AC staining was conducted as described above omitting the nickel enhanced TH antibody staining steps. After staining, brain sections were washed and stored in PB, mounted and cover-slipped.

### 2.6. Electrophysiological mapping

Four additional female mice, between 12 and 14 weeks of age, were used to determine the alignment between electrophysiological and anatomical atlas maps, which were used as the basis of identifying tissue sections for AC staining. Auditory brainstem response (ABR) and cortical electrophysiology were performed following protocols described in previous publications (Shepard et al., 2015b). Briefly, peripheral hearing thresholds were assessed via ABR for clicks and tone pips at 8,16, 24, 32, 64 and 80 kHz (3 ms, 21 Hz repetition rate) to ensure animals were responsive to auditory cues. Frequency responses were next mapped electrophysio-logically across the left hemisphere’s AC. Multiunits were recorded across a craniotomy over the left AC using a 4 MΩ 3 × 1 tungsten matrix microelectrode (FHC) with 305 μm interelectrode spacing. The electrode array was driven into layer 4 (400 μm), and pure tones were played back (60 ms, seven intensities from 5 to 65 dB SPL, 30 frequencies log-spaced 2–32 kHz) in pseudorandom order, with five repetitions of each frequencye–intensity combination. Stimuli were presented with a free-field speaker positioned 20 cm from and 45 anterolateral to the right ear.

To identify the spatial extent of core AC, consisting of primary auditory cortex (A1), anterior auditory field (AAF), and ultrasound field (UF), we looked for clear frequency tuning and a peristimulus time histogram (PSTH) peak <40 ms from sound onset, and in the case of UF, we looked for best frequencies (BF) > 50 kHz (Stiebler et al., 1997; Shepard et al., 2015b). Sites at the cusp of the A1-AAF best frequency gradient reversal point were denoted as “AAFA1.” Weak or absent PSTH peaks, or latencies >40 ms indicated that a site was outside core AC, belonging instead to ventral secondary (A2) or dorsal posterior (DP) auditory fields. BFs were defined as the frequency that generated the highest average spike rate over all the intensities equal to or less than the threshold intensity. Individual animals’ BF maps were generated by Voronoi tessellation (Matlab: voronoin) of all recording sites for a given animal.

After electrophysiological mapping, brains were electrolytically lesioned at dorsal and ventral sites located in different rostralcaudal locations within AC (A365 World Precision Instruments, Inc. 2.5uA 5s), followed by perfusion with 4% paraformaldehyde for tissue fixation. The tissue was then preserved in 30% sucrose before being sectioned at 40 mm thickness on a microtome. Sections were stained in an alternating fashion with Nissl, DAB parvalbumin (PV, Swant PV235 Mouse monoclonal 1:4000), and DAB calbindin (CB, Sigma C9848 Mouse monoclonal 1:4000) repeating every 3 sections. Lesions were localized on the sections and matched to a cortical surface image and penetration map of the AC generated during electrophysiological experiments. Each section was aligned to the cortical surface image to scale based on the spacing of each PV-stained or CB-stained section (every 3rd section, each with 40 μm spacing, corresponding to a total of ~120 μm spacing between sections). Expected spacing of lesions based on the cortical surface image was confirmed in sections. Relative sizes of each physiologically determined auditory region (A1, AAF, AAFA1, A2, UF, DP, as determined by a combination of BF and response onset latency) on each section were estimated from the relative pixel conversion of the size of each region found on the corresponding cortical surface image.

### 2.7. Quantification

**Fig. 2.**
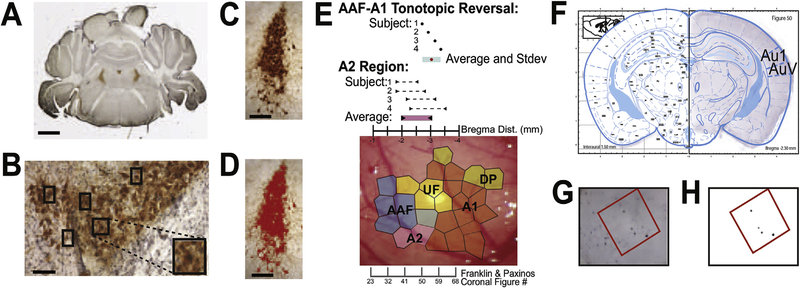
TH/c-Fos immunohistochemistry, quantification and methodological steps for the cortical c-Fos-IR neuron counting in the AC. **(A)** Whole-slice image of TH (brown) staining in the LC. Scale bar is 1 mm. **(B)** Double stained LC where TH-IR brown staining localizes in cell bodies and c-Fos-IR stains some nuclei purple. Boxes indicate TH-IR/c-Fos-positive co-stained cells. Scale bar is 10 μm. **(C)** Before and **(D)** after color-thresholded image of TH stained cells used to measure area of LC. **(E)** Electrophysiological mapping and atlas alignment image over auditory cortex for mapping and auditory region assignments. Coronally sectioned brains were aligned to mouse atlas (Franklin & Paxinos, 3rd ED). Bregma coordinates of the brain section at which the AAF-A1 transition occurs over four individual animal subjects: Red: mean bregma location of transition (−3.035 mm); Blue: Mean ± Stdev (−3.3218~−2.7482 mm); Black: subject animals (n = 4). Bregma Coordinates of A2 – Average Rostral ~ Average Caudal Bregma (mm): Pink: rostral ~ caudal average −2.03~ −2.94 mm), black arrows: individual animals (n=4). Voronoi tessellations overlaying example image of cortex delineate auditory cortical area assignments. **(F)** Example of an immunohistologically stained section scanned at 4 × and overlaid with the best matched atlas _fi_gure image (Franklin and Paxinos, 2013). **(G)** 20× images with 200× 200 μm square area at the center of the cortical area of interest selected for thresholding **(H)** Thresholded 200 × 200 μm square identifying neurons with c-Fos-IR.

Protein expression in the LC was quantified in 6 ± 2 tissue sections per animal, spanning the full histological depth of the LC, verified by TH staining. TH staining clearly delineated the LC at approximately 5.34 mm–5.80 mm caudal to Bregma (Fig. 2A). Using a light microscope (Zeiss Axioplan) with an attached camera, images of the LC were taken with a 20 × objective in the imaging program, MagnaFire. Using the 40 × objective of the light microscope, c-Fos-expressing TH-immunoreactive (TH-IR) cells were quantified by eye in the LC by an observer blind to treatment condition. TH and c-Fos costained cells were identified as a TH-IR stained cell body surrounding a purple c-Fos-positive stained nucleus (Fig. 2B). The density of brown TH-staining of cell bodies precluded the quantification of individual TH-stained neurons, therefore the area covered by TH-IR cells in each section was manually color thresholded and measured to include all cells with TH-IR using NIH Image J (Fig. 2C). The area of TH-IR was measured at 20 × in square pixels and converted to square millimeter. The number of c-Fos-positive TH-IR cells, which were clearly discernible as niDAB-labeled nuclei, was expressed as a proportion of the thresholded LC area.

Coronal AC sections were identified at 2× using a slide imager (Meyer Instruments Path Scan Enabler IV slide imager). Both hemispheres of each section were matched to the best representation of its cortical position using The Mouse Brain atlas (Franklin and Paxinos, 2013) and overlaid with the matching atlas figure using Adobe Photoshop CS6 (Fig. 2F). The Mouse Brain atlas presents sequential coronal sections as numbered figures, and the convenient asymmetry of the hemispheres in each figure allow for close matching between stained sections and hemisphere specific atlas images. Each altas hemisphere containing Au1 and/or AuV was assigned specific rostral-caudle positions on an ordinal scale. The hemispheres of each 2× stained section image were overlaid with the best matching atlas figure. This aided in 20× magnification imaging of Au1 and AuV using a light microscope (Zeiss Axioplan).

The center of the cortical region of interest was identified and layers V/VI imaged using a light microscope with an attached camera and Magnafire software. Perpendicular and at least 50 μm distal from the floor of layer VI, a 200 × 200 μm^2^ square area was selected within an Atlas defined cortical brain region. To ensure unbiased stereological methodology, an observer blind to condition assigned the square location, with no regard to cell expression and conducted this analysis.

Layers V/VI were chosen for analysis because estrogen receptors have been primarily found in these deeper layers in mouse AC (Charitidi and Canlon, 2010), and thus we anticipated the greatest effect of E2 may occur here. Moreover, layers V/VI of both the primary and secondary auditory cortices send output projections to subcortical and other cortical sites (Llano and Sherman, 2008), making them particularly relevant for characterizing auditory cortical interactions with other brain areas.

ImageJ software was used to manually threshold our 200-μm^2^ squares using the Yen filter, process for noise smaller than 9 pixels and count remaining objects to determine number of c-Fos-IR neurons. Imperfections such as small bubbles or debris within the count area prompted counting by eye. AC counts were converted to densities in cell/mm^2^ to facilitate consistent statistical analyses of both LC and AC.

Auditory cortical sections for cell quantification were chosen using our electrophysiological boundaries as well as The Mouse Brain atlas (Franklin and Paxinos, 2013). Stained sections were matched to the best figure and hemisphere of the atlas (See above). The dorsal and ventral boundaries of both the primary and secondary auditory cortical regions were determined using the atlas. The rostral and caudal extent of AAF to AI used were those of the atlas’ Au1, i.e. from bregma −2.2 mm to −3.6 mm or atlas Figs. 49–61, similar to previous publications (Tsukano et al., 2015). We found the mean bregma coordinates of the physiological AAF-AI transition occurred at −3.035 mm with a standard deviation of −3.3218~−2.7482 mm (Fig. 2E), comparable to previous publications that also used bregma positions within this range (Llano and Sherman, 2008; Tsukano et al., 2015). The averaged rostral to caudal bregma coordinates of the physiologically determined A2 extended between −2.03 mm and 2.94 mm, or the left hemisphere of atlas Fig. 53 to the left hemisphere of Fig. 58 in The Mouse Brain atlas. This alignment of the physiological auditory cortical fields to the atlas thus specified the best sections to designate as the AC core, AI-AAF high frequency transition area (AC-hf) and secondary (A2) auditory regions for c-Fos-IR cell counts. We use the AC core, AC-hf and A2 terminology when describing c-Fos-IR results.

### 2.8. Behavior analysis

A subset of video recorded from the 10-min playback of pup-calls was subjected to behavioral analysis. Video tracking was performed with TopScan (CleverSys Inc). Video preprocessing involved image distortion correction and calibration of arena to the 171.45 mm length of the cage. A text file was generated containing positional data captured at 30 frames per second; this was the input for MATLAB functions written to analyze movement. For each video, average velocity was determined by creating a vector with frame-by-frame pixel displacement of the animal’s center-of-mass. To account for errors in motion tracking, a 20th order one dimensional median filter was applied to the vector. Quadrant crossings were counted in MATLAB, based on the XY coordinates of the middle of the cage. Percentage of time spent stationary was determined by evaluating the distance travelled for each second. If the distance was within a radius of 1 mm, it was considered to be random image jitter, and that one second period was marked as stationary. While using awake and freely moving animals can make it difficult to unambiguously assess attention to or arousal by the stimulus, we used movement during sound playback as a proxy.

### 2.9. Statistical analysis

Statistical analyses were performed in JMP data analysis software, unless otherwise stated. For each of the three brain regions and three behavior measures, the Huber Robust Fit Outlier application was used to identify cell count per section and behavioral outliers. Of the 423 LC, 354 AC core and 345 A2 section cell count and area measurements, 13, 2 and 3 were omitted for data analysis respectively. The LC outliers contained counts from all four groups (6 E/CC, 2 B/CC, 3 E/N, and 2 B/N). In the behavioral analysis, one animal was identified as an outlier with the Huber Robust Fit test for Average Velocity and Quadrant Crossing. Video of this outlier’s behavior showed perpetual circular running motion throughout the 10-min pup-call playback. This animal was not sacrificed for cFos-IR and based on its outlier status and unusual behavior, it was removed from all behavior analysis.

Two-way ANOVA was conducted for each brain area. To calculate the average LC c-Fos-IR per animal, first the sum of all an animal’s LC section counts was divided by the sum of the animal’s calculated LC area. Second, this was multiplied by the pixel to mm conversion to produce an average LC-IR per animal. This method did not overly represent sections with high cell counts and small areas. Chi-squared test was used to compare the LC c-Fos-IR of animals, which experienced playback or background stimuli. AC core and A2 averages per animal were calculated by averaging all an animal’s cell counts per 200 μm^2^ and converted to c-Fos-IR per mm^2^. Tukey’s post-hoc analysis was used, unless the Levene test indicated a significant difference in variance across groups, in which case Steel-Dwass was run instead.

To determine how our experimental conditions influence neural activity, we applied a Generalized Linear Mixed Model and considered that c-Fos-IR would depend on brain region, treatment group as well as nuisance parameters associated with an individual animal. Our statistical model treats our c-Fos-IR count observations as (quasi−) Poisson distributed, with rate parameter given by:

$$n\sim \text{Poisson}(\lambda )\text{log}\lambda_{\text{rai}}=\beta_{\text{r}0}+{\text{x}}_{\text{ha}}\beta_{\text{rh}}+{\text{x}}_{\text{ea}}\beta_{\text{re}}+{\text{x}}_{\text{ha}}{\text{x}}_{\text{ea}}\beta_{\text{r},\text{h}\times \text{e}}+\text{log}{\text{A}}_{\text{i}}$$

where *x* is the treatment value (0 or 1), *β* is effect magnitude, *A* is sample area, and the indices are *r* for brain region, *a* for animal, *h* for hormone, *e* for experience, and *i* for section ID. Unlike the two-way ANOVA analysis, no average per animal values were used here, and instead individual section counts and areas were labeled by their animal’s ID. Using a quasi-Poisson distribution and mixed modeling, the covariance due to samples coming from the same animal was directly estimated, strengthening the statistical power compared to per-slice pooling or per-animal total pooling analysis. The cross-region covariance was determined by plotting the peranimal random effects for two regions.

## 3. Results

We sought to determine whether the way in which the neurochemical E2 modulates the noradrenergic LC and the AC when listening to conspecific vocalizations is affected by prior experience learning the social relevance of the sounds. Adult female mice aged 10.6 ± 0.83 weeks (mean ± standard deviation) were ovariectomized and assigned to 1 of 4 animal groups. We used a 2 × 2 factor experimental design to investigate how pup care experience (N vs. CC) affects the expression of the IEG c-Fos in response to the playback of pup isolation calls, while controlling for systemic estrogen (E2 vs. B). Animals were exposed to a pup-call sound stimulus at 13.4 ± 0.9 weeks, 19 ± 4 days after hormone (or vehicle) implant surgery. We measured c-Fos-IR in the LC, as well as in cortical areas including the typical physiological extent of AC core, a high frequency AC subregion and A2, to characterize the response and relationship between these brain regions to novel or familiar social auditory stimuli.

### 3.1. Differential impact of experience and hormone on pup-callinduced LC and AC c-Fos-IR

**Fig. 3.**
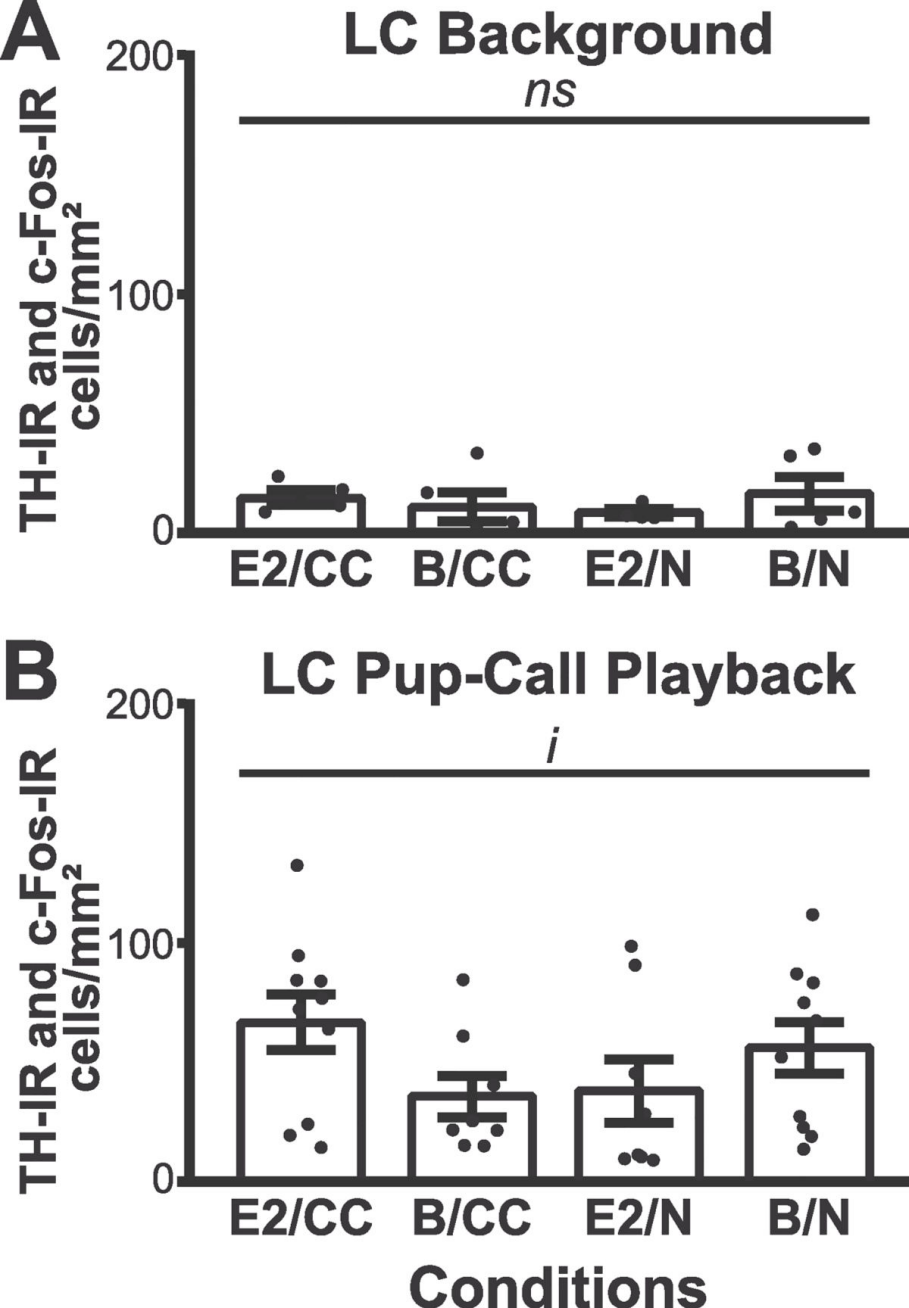
LC c-Fos-IR expression varies between stimuli, with pup-call playback producing differences in expression dependent on pup experience and hormone treatment. C-Fos-IR per mm^2^ in relation to pup experience and hormonal state conditions. **(A)** LC response to playback of sound stimuli filtered to remove pup-calls (background). No significant difference between groups. **(B)** LC c-Fos-IR in response to unfiltered sound stimuli containing pup-calls, showed an interaction between pup experience and hormone state but no main effects. *ns*, not significant; *i*, interaction, p < 0.5.

We quantified c-Fos-IR using multiple tissue sections from the three brain regions of each animal (see Section 2.7). We determined that a sound stimulus containing background noise without pupcalls did not modulate LC expression of c-Fos-IR across our animal groups (*F*_1,34_ = 0.45, *p*= 0.715), where expression levels were overall fairly low (Fig. 3A). However, there was a significant difference between the LC c-Fos-IR of animals who experienced background sound playback and those who experienced pup-call playback (χ^2^ (1) = 19.39, *p* < .0001, not shown). In the LC of animals hearing pup-calls, there was no main effect of experience (*F*_1,34_ = 0.64, *p* = 0.21) or hormonal treatment (*F*_1,34_ = 0.44, *p* = 0.51) of c-Fos-IR. However, there was a significant interaction between the two factors (*F*_1,34_ = 5.20, *p* = 0.029) (Fig. 3B), indicating LC activity following pup-call exposure depends on both hormonal state and prior pup experience. No post-hoc significant differences between groups were found.

In quantifying AC expression, we first examined sections taken over the entire rostral-to-caudal extent of the anatomically defined AC core region, averaging cell counts for each animal (1.46 mm span, see Methods). Here, we found a main effect of experience leading to a decrease in c-Fos-IR (*F*_1,12_ =12.54, *p* = 0.004), but neither a main effect of hormone (*F*_1,12_ = 2.05, *p* = 0.17) nor an interaction between hormone and experience (*F*_1,12_ = 0.10, *p* = 0.76).

**Fig. 4.**
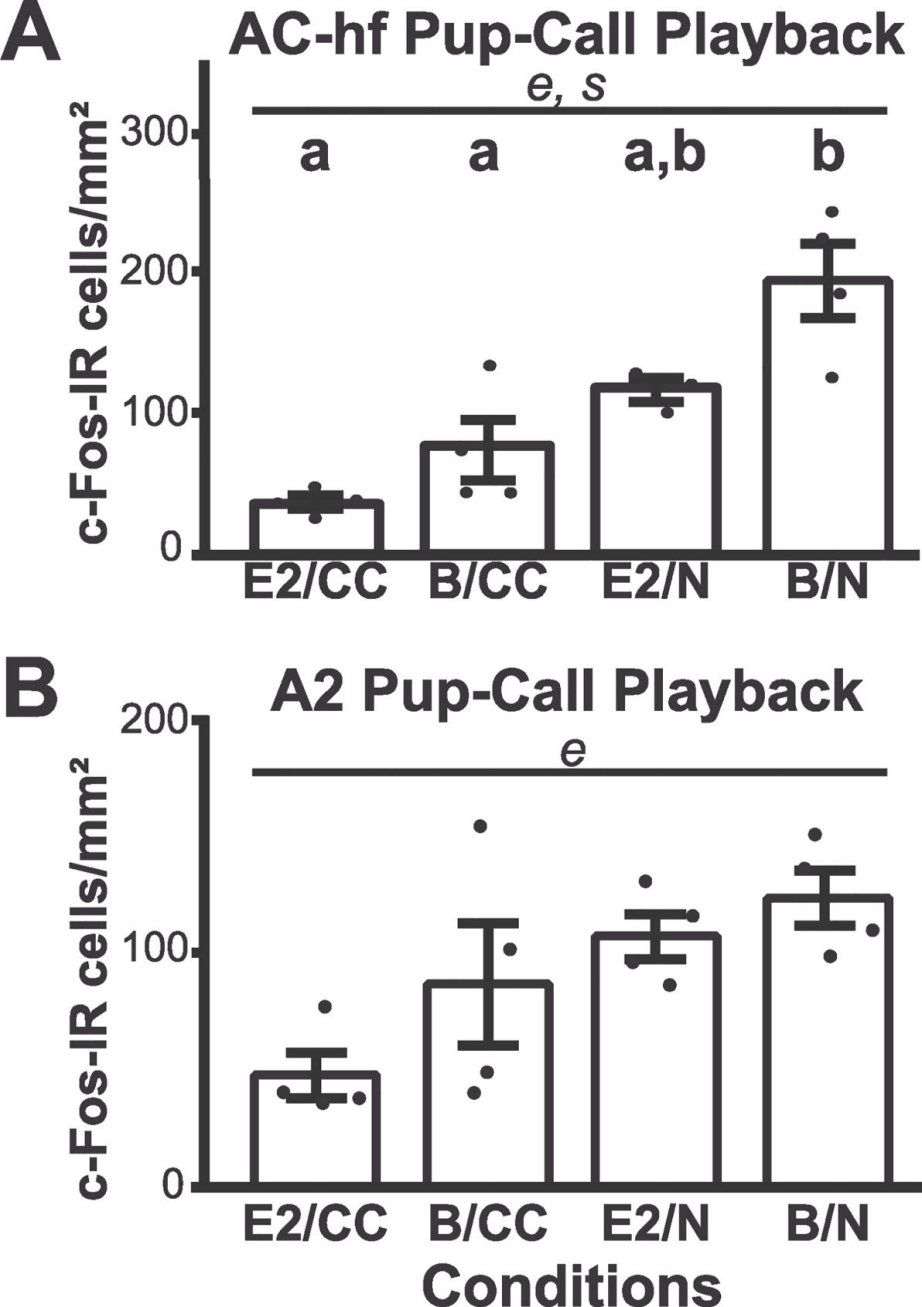
C-Fos-IR expression in response to pup-call playback varies within the Auditory Cortex, and depends on pup experience and hormone treatment. C-Fos-IR per mm^2^ in relation to pup experience and hormone exposure conditions. **(A)** AC-high frequency region and **(B)** A2. **(A)** In the AC-hf region there was an effect of experience. Both in the AC-hf region and **(B)** in the A2 there is a main effect of experience. Points indicate average c-Fos-IR in 1 mm^2^ and bars indicate standard error. *e*, experience, p < 0.5; *s*, hormonal state, p < 0.5.

Previous research suggested a region within the AC core, near the point of tonotopic map reversal between A1 and AAF where neurons are particularly sensitive to vocalizations (Issa et al., 2014). Thus, we also performed a restricted analysis on this physiologically validated (see Section 2.4) high frequency region of AC core (AC-hf). In the AC-hf we saw a significant decrease in c-Fos-IR, associated with a main effect of pup-care experience (*F*_3,12_ = 28.43, *p* = 0.0002). Additionally, a main effect of E2 treatment (*F*_3,12_ = 9.33, *p* = 0.011) emerged (Fig. 4A). Unlike the LC, there was no interaction between hormone and experience (*F*_3,12_ =1.22, *p*= 0.29), and instead, it appeared that both E2 and pup experience tended to reduce c-Fos-IR. Indeed, post-hoc analysis found a significant difference between E2/CC and B/N animals (*p* = 0.0003, TukeyKramer) in this AC-hf region, as well as between B/N and B/CC animals (*p* = 0.002, Tukey-Kramer). Thus, five days of social experience, and to some extent hormone state, led to decreased c-Fos-IR in response to social auditory stimuli in the AC-hf subregion.

We next quantified animal-averaged c-Fos-IR over a physiologically validated A2 region (Fig. 2E). We found a main effect of experience (*F*_1,12_ =11.91, *p* = 0.004), but not of hormone (*F*_1,12_ = 2.98, *p*= 0.10). There was also an absence of an interaction (*F*_1,12_ = 0.36, *p* = 0.55) (Fig. 4B). Unequal variance between groups (p = 0.03, Levene) can be attributed to the significance high variance in B/CC group’s c-Fos expression, necessitated non-parametric post hoc tests that found no significant post-hoc differences (SteelDwass). Nevertheless, five days of social experience led to the decrease in c-Fos-IR in the A2, as in AC core and AC-hf. Notably, out of the three regions examined, the effect of experience was the greatest in the AC-hf.

**Fig. 5.**
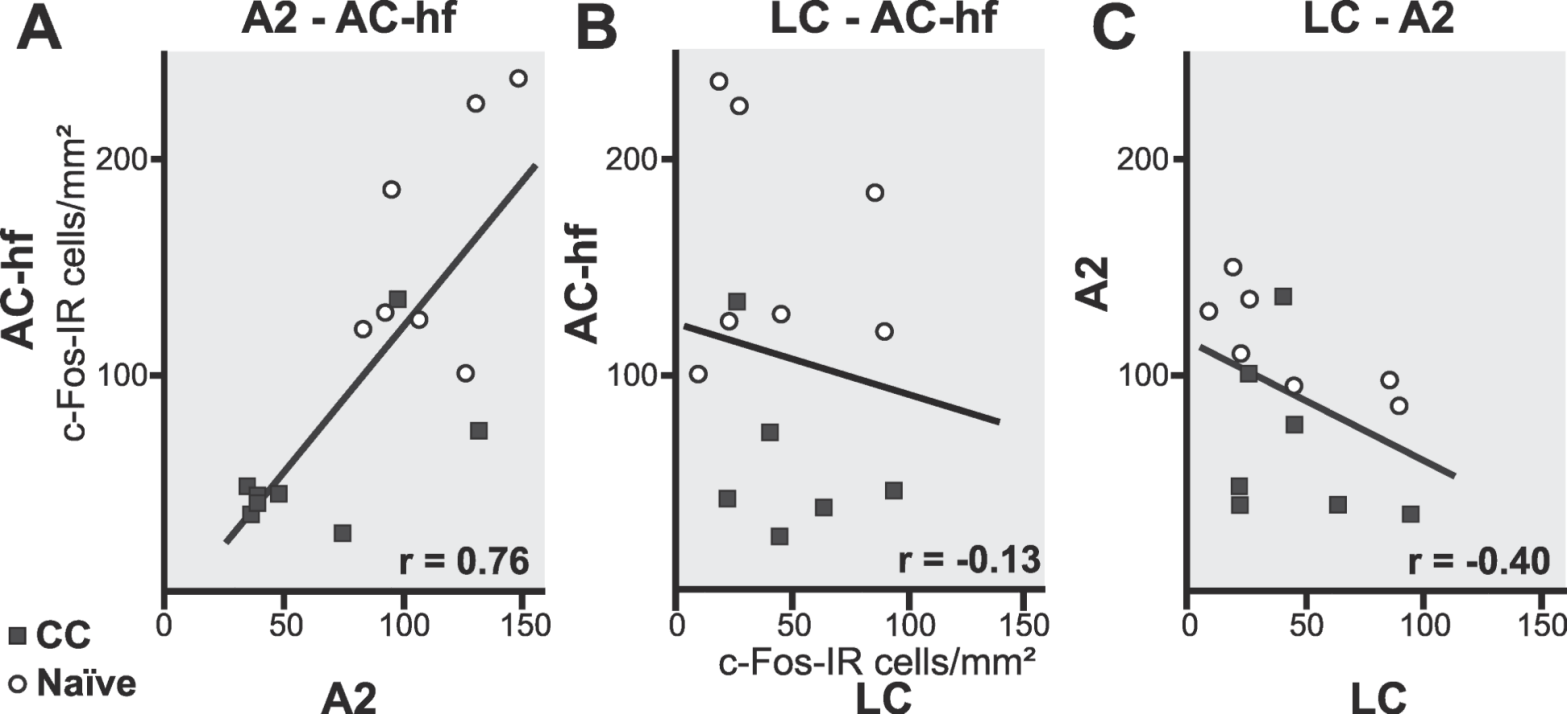
Correlation between brain regions. C-Fos-IR expression averages per animal and brain region with solid line being best fit; **(A)** AC-hf and A2 (r = 0.76, p = 0.0009) **(B)** AC-hf and LC (r = 0.13, p = 0.64) and **(C)** A2 and LC (r = −0.40, p = 0.14).

Lastly, we collected and analyzed sections from all three brain regions in a subset of animals to consider the cross-region covariance (Fig. 5). There was a high correlation between the two auditory cortical areas, AC core and A2 (r = 0.83, p < 0.0001, not shown) and between the AC-hf and A2 (r = 0.76, *p* = 0.0009, Fig. 5A), agreeing with the expectation that sound-driven AC core and A2 activation in the same animal should be related. There was little to no correlation between LC and AC core (r =− 0.21, *p* = 0.46, not shown) or AC-hf (r =− 0.13, *p* = 0.65, Fig. 5B), and a non-significant negative correlation between LC and A2 (r =−0.40, *p* = 0.14, Fig. 5C).

### 3.2. GLMM analysis confirms differential effects of experience and hormone on LC and AC

The analyses above relied on c-Fos-IR expression averaged within animal, reducing the ability to leverage the statistical power of multiple observations within an animal. To further confirm the strength of our results, we next applied a GLMM (see Section 2.9) to the data from each tissue section. The mixed model accounted for sampling differences between brain regions, the fact that observation from the same animal are not independent and considered individual animal variation in overall c-Fos-IR expression. Our measurements were standardized as cells per mm^2^ in the AC-hf, A2 and LC. B/N animals were set as the baseline, and the analysis was bootstrapped with 1000 resamples (Table 1). In situations with several experimental variables, this approach can yield higher statistical power than traditional methods that make it difficult to account for numerous sources of experimental variance (Li and Dye, 2013).

**Fig. 6.**
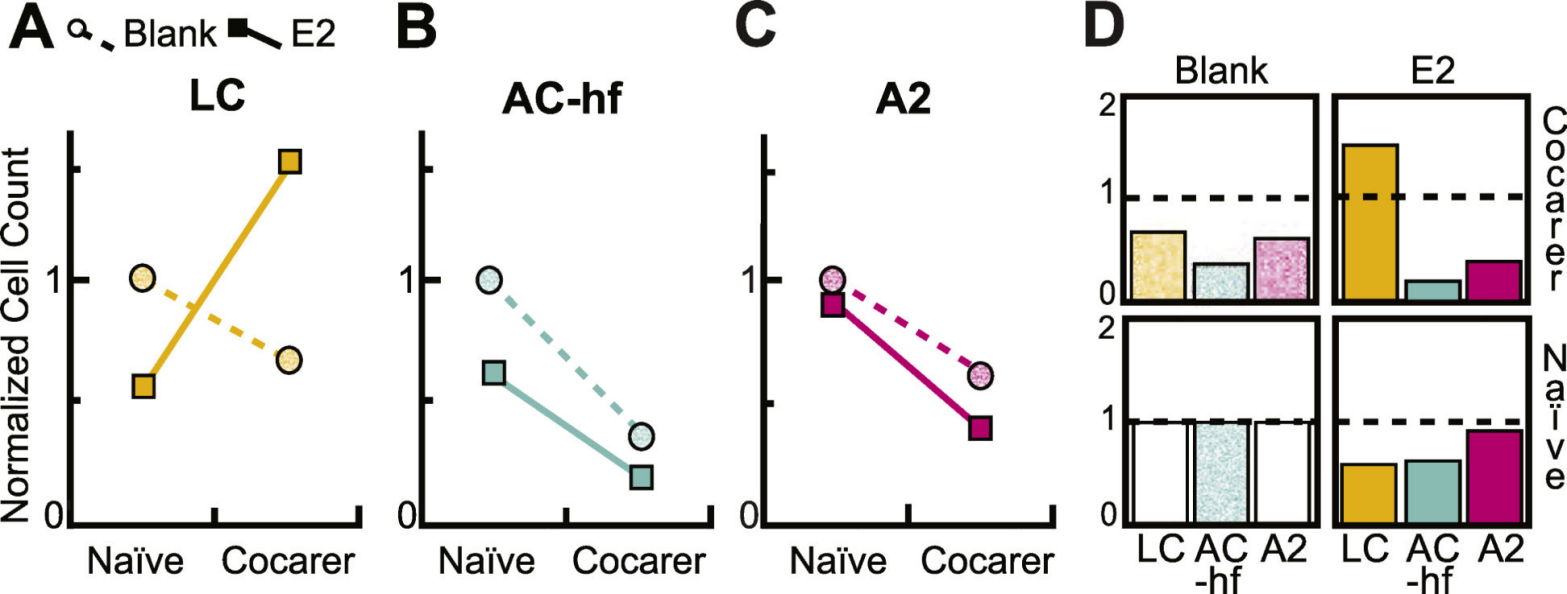
GLMM analysis verifies majority of ANOVA findings. **(A)** In the LC, there is an interaction between pup experience and hormone state (*p* = 0.008) but no main effects. **(B)** The Au1-high frequency region displayed a robust main effect of experience (*p* = 6.55e^−06^) signi_fi_cantly decreasing c-Fos-IR with experience. **(C)** The A2 there was a main effect of experience. A2 c-Fos-IR per mm^2^ also decreases with pup experience (*p* = 0.020). **(D)** Bar graph representation of normalized results indicates c-FosC-fos-IR in each of the three brain regions for the 2 × 2 experimental conditions. Normalized to blank/naïve condition values and set at 1 (represented by dashed line).

As with the animal-averaged ANOVA analysis, the GLMM found a significant interaction between E2 availability and pup experience in the LC (*p* = 0.008), as demonstrated by the crossed-lines in Fig. 6A showing the normalized cell counts. Further supporting the ANOVA findings, the GLMM also found decreases in c-Fos-IR expression as a main effect of pup experience in the AC-hf (*p* = 5.50^−6^), and in A2 (*p* = 0.018) (Fig. 6B–C). A main effect of hormone on AC-hf expression, reported in the ANOVA analysis, was trending (*p* = 0.093), as was a similar main effect on LC (*p* = 0.058), suggesting a weak direct hormone effect in these brain areas.

### 3.3. Differential effects of experience and hormone on behavioral response to pup-call playback

In a subset of animals (n = 38), videos of the 10-min playback session were analyzed to assess coarse behavioral differences across groups in response to pup-call stimuli playback. The behaviors analyzed included the percentage of time spent stationary, average velocity, and number of quadrant crossings. All three behaviors showed significant interaction between hormone treatment and pup-experience.

**Fig. 7.**
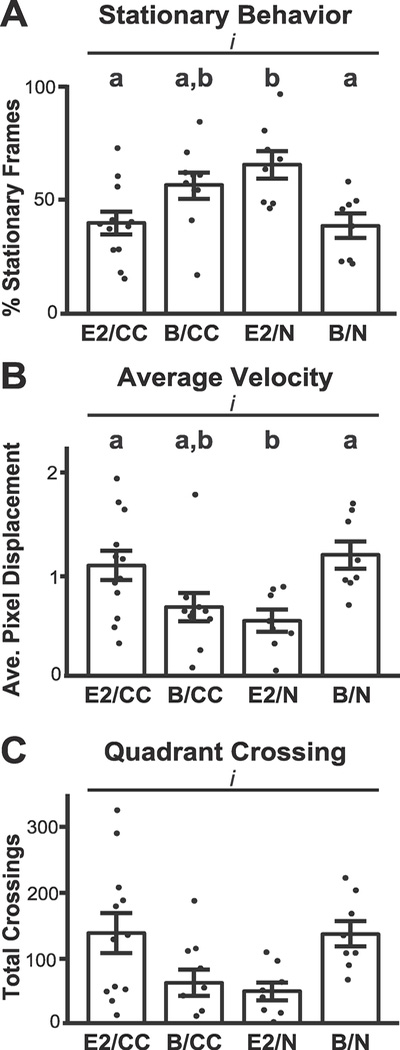
Stationary behavior and LC c-Fos expression. **(A)** Percent stationary frames per condition. Bars are standard error. No significant difference between social experience and hormone state. **(B)** Multivariate analysis of stationary behavior and c-Fos-IR found that r =−0.70, p = 0.015 with outlier removed (X). *, p < 0.5.

Although data on the percentage of time spent stationary (Fig. 7A) revealed no significant effect for experience (*F*_1, 35_ = 0.53, *p* = 0.47) or hormone (*F*_1, 35_ = 0.83, *p* = 0.36), we found a significant interaction between experience and hormone (*F*_1, 35_ =15.63, *p* = 0.0004). E2/CC and B/N groups showed the lowest levels of stationary frames. Post-hoc analysis showed a significant difference within E2 treatment groups (E2/N versus E2/CC, *p* = 0.009, Tukey-Kramer), as well as a significant difference within the naïve condition groups (E2/N versus B/N, *p* = 0.01, Tukey-Kramer).

When animals were moving, an analysis of average velocity (Fig. 7B) found no significant main effect of experience or hormone (*F*_1, 34_ = 0.01, *p* = 0.90; *F*_1, 34_ = 0.68, *p* = 0.41). Yet like stationary behavior, this behavior measure displayed a significant interaction between experience and hormones (*F*_1, 34_ =14.25, *p*= 0.0006). Significant post hoc differences were present within the E2 treatment groups (E2/N versus E2/CC, *p* = 0.03, Tukey-Kramer), as well as within the naïve condition groups (E2/N versus B/N, *p* = 0.02, Tukey-Kramer). The quadrant crossings data (Fig. 7C) also revealed no significant effects for experience or hormone (*F*_1, 34_ = 0.53, *p* = 0.47; *F*_1, 34_ = 0.47, *p* = 0.40) but the behavior was significantly affected by an interaction of E2 and pup experience (*F*_1, 34_ =11.52, *p* = 0.001). Number of quadrant crossing did not show significant post hoc differences, though there was a trend towards significance within the E2 treatment group (E2/N versus E2/CC, *p* = 0.055, Tukey-Kramer).

The interaction between experience and E2 treatment for our three behaviors resembled the interaction we found in LC c-Fos-IR (Fig. 3B), though correlations between behavioral measures and LC c-Fos-IR for the smaller group of animals where we obtained both data (n = 12) did not reach significance (stationary behavior r =−0.45, *p* = 0.13; average velocity r = 0.46, *p* = 0.12; quadrant crossing r = 0.43, *p* = 0.15).

## 4. Discussion

Our work used an ethological learning context (Bennur et al., 2013) to study how the sensory plasticity-related, noradrenergic neuromodulatory area LC is engaged as a function of prior experiences and internal hormonal state. We exploited a mouse model of maternal recognition of pup-calls, which arises through experience caring for pups and is facilitated by the hormone E2 (Stolzenberg et al., 2009; Koch and Ehret, 1989). Our manipulation of E2 availability and pup care experience in virgin females allowed us to model the physiological and experiential state of motherhood (Miranda et al., 2014) to test how these factors affect neural responsiveness in both LC and AC to infant vocalizations when they are either novel or familiar.

When we exposed female mice to the sound of pup vocalizations, but not a sound containing behaviorally irrelevant background noise, we found prior experience caring for pups and the presence of maternal levels of E2 interacted to alter the expression of the plasticity-related IEG, *c-fos*, in noradrenergic LC neurons. Estrodiol-treated females with pup experience (E2/CC) and naïve females without estradiol (B/N) showed the highest LC c-Fos-IR, average velocity behavior and number of quadrant crossing behavior as well as the lowest amount of stationary time during the pup call playback. Familiarity with pup-calls significantly decreased the c-Fos-IR expression in both AC core and A2, especially in the central AC-hf region, previously linked to vocalization processing (Tsukano et al., 2015; Issa et al., 2014). Thus, far from being stereotyped and hard-wired, the brain’s response to natural vocalizations is situationally dependent on hormonal state and prior familiarity with the vocal category. Below we interpret our results in light of the current literature on LC and AC, placing them in the context of a working model wherein a social stimulus’ familiarity leads to the genomic activation of a reduced subset of auditory cortical neurons compared to when the sounds are initially novel.

### 4.1. LC processing of infant cues

We found playback of the pup-calls, but not background noise, led to elevated LC c-Fos-IR, indicating that species-specific infant vocalizations engage this key neuromodulatory area implicated in cortical plasticity (Edeline et al., 2011; Martins and Froemke, 2015). This result is consistent with the importance of the neuromodulator NE in maternal learning and behavior. Dams lacking the enzyme dopamine beta-hydroxylase, which synthesizes NE from dopamine, fail to exhibit maternal responsiveness (Thomas and Palmiter, 1997). Curiously, restoring the ability to synthesize NE in these knock-out animals by administering before first parturition a synthetic precursor for NE production reestablishes maternal behavior for the first litter and subsequent litters, suggesting that NE not only affects initial maternal responsiveness but also maternal memory (Scanlan et al., 2006; Levy et al.,1990). That such a memory could involve NE-mediated experience-dependent plasticity for the sensory cues associated with infants has been suggested previously (Miranda and Liu, 2009; Banerjee and Liu, 2013).

We further found the degree of LC genomic activation by pup-calls had a complex sensitivity to the systemic availability of the hormonal steroid E2 and whether the calls carried behavioral significance from prior pup care experience (Figs. 3 and 6). C-Fos expression is facilitated by phasic firing (Labiner et al., 1993; Dragunow et al., 1989), which is induced in LC by arousing stimuli (Devilbiss and Waterhouse, 2011; Martins and Froemke, 2015). Our significant interaction between experience and hormones in LC c-Fos-IR suggests our animal groups were aroused differently by the identical stimulus, a result reinforced by our behavioral analysis (Fig. 7). On the one hand, although pup-calls can be arousing when novel, inducing higher LC c-Fos-IR in blank-implanted naïve mice, elevated systemic E2 dampens this response, consistent with E2 reducing general stress and hyperactivity (Tang et al., 2005; Daendee et al., 2013). On the other hand, while calls are familiar and perhaps less arousing to blank-implanted cocarers, E2 heightens their arousal and LC c-Fos-IR, potentially reflecting an ability of reproductive hormones to enhance maternal responsiveness to previously experienced pup cues (Fleming and Sarker, 1990). Notably, the contrast in LC’s versus AC’s pattern of c-Fos-IR expression across groups further reinforces the conclusion that the auditorineural drive is not the only determinant of LC response, which also depends on both internal physiological factors and prior experience.

The prior experience we highlighted here was pup care, but it should be noted that our housing condition also introduced a separate experiential factor that could potentially impact our findings. Specifically, our naïve animals were individually rather than socially housed in the days between ovariectomy and sound exposure, unlike our cocarers who were housed with mothers and their pups. Even though long-term social isolation in mice can lead to abnormalities such as more anxiety-like behavior, greater hyperactivity, impaired recognition memory and changes in social interactions (Valzelli, 1973; Koike et al., 2009; Voikar et al., 2005) our naïves’ social isolation alone likely cannot explain their pattern of behavioral and LC c-Fos-IR results. First, social isolation effects on behavior are usually studied after at least 4-weeks or more of isolation after weaning e longer than the period that our naïve, adult animals were isolated. Second, even though c-Fos expression in socially isolated rodents is associated with increased levels of catecholamines such as norepinephrine (Heritch et al., 1990), we did not see a baseline elevation of average LC activation in naïve vs. cocarer animals hearing background noise (Fig. 3A). Third, pup-call playback also did not change the average LC activation between these two groups, which might have been expected based on social isolation effects on c-Fos in other brain areas after brief social encounters (Ahern et al., 2016). Hence, our findings of significant experience and hormone interaction rather than main effects on LC c-For-IR likely do not arise from our naïve subjects’ social isolation.

### 4.2. Experience-dependent changes in AC

As noted above, we found c-Fos-IR in AC (Figs. 4 and 6) had a strikingly different dependence on hormones and experience compared to LC. Familiarity with a social auditory cue was associated with decreased c-Fos-IR in both auditory cortical sites examined. Previous literature has differed on whether familiarity with a cue increases or decreases c-Fos expression within the AC. Rats listening to complex, non-social sounds show no difference in the primary AC c-Fos-IR between novel and familiar sound playback while the secondary associated area displays a significant decrease in c-Fos-IR when the sound is familiar (Wan et al., 2001). On the other hand, in the ultrasonic vocalization literature, stimulation with pure tone models of pup-calls induce lower levels of c-Fos-IR in the ultrasonic field of the core AC in dams compared to naïve females, while the former expresses higher secondary AC c-Fos-IR than the latter (Fichtel and Ehret, 1999). The A2 response was associated with the “news-worthy” nature of the stimuli (Geissler et al., 2016). This discrepancy with our results for A2 may be due to our use of natural pup-calls, which, while technically novel, potentially sound more acoustically familiar to dams than tonal models of calls, and thus be less “news-worthy.”

In considering our AC c-Fos-IR results, it is important to note that animals were free to move within the cage, and thus could have experienced different sound levels for calls during the playback. Despite this caveat, which undoubtedly creates some variability across animals, it is unlikely that variation in sound level from the positioning of the animal could explain the large, systematic group differences in auditory cortical responses (Fig. 4). In fact, movement is not correlated with AC c-Fos expression, as is particularly obvious for E2/CC vs. B/N animals (compare Figs. 7–4), showing similar average movement, but vastly different AC c-Fos.

Mouse pup communication sound processing is believed to be lateralized to the left hemisphere (Geissler and Ehret, 2004; Ehret, 1987; Marlin et al., 2015). Here, we did not consider hemispheric cell expression in the collection of our data, but the persistence of a significant effect of pup experience despite this homogenization speaks to the robustness of our observed effects. Within core AC, an AC-hf sub-region has been linked to species-specific vocalization processing in mice (Issa et al., 2014; Tsukano et al.). In fact, Tsukano et al. delineate a part of this transition area as a dorsomedial subfield that is particularly sensitive to ultrasonic vocalizations. Our finding that pup familiarity effects on pup-call induced responses were highest in this region, which we delineated through alignment to electrophysiological maps (in separate animals), adds further support for this region’s heightened importance within AC for communication sound processing. Further research is needed though to determine whether this area specifically processes vocalizations such as regions identified in the human AC (Belin et al., 2000), or one that is activated by ultrasonic vocalizations simply because of its high frequency responsiveness.

That experience with pups consistently reduces pup-call induced c-Fos-IR in core AC may seem counter-intuitive, especially if increased AC activation is presumed to underlie sound recognition. In fact though, expansion in the area of auditory cortex responding to a newly behaviorally relevant signal is not observed as pup-calls gain significance (Shepard et al., 2015b). Instead, one should consider that sensory cortices may balance stable representations of previously learned stimuli against plasticity for newly relevant stimuli. Since our pup experienced animals had five-days of experience with vocalizing pups by the time we exposed them to call playback, these subjects may have already experienced substantial cortical plasticity to optimize pup-call recognition. Since c-Fos expression not only reflects neural activation but also active cellular mechanisms for learning (de Hoz et al., 2017; Mayer et al., 2010), c-Fos-IR would then be low in response to the already familiar auditory cues. Diminished production of a plasticity associated protein in response to a familiar stimuli, that previously gained meaning through social experience, might be reflected in a reduced subset of neurons constituting the most behaviorally useful circuit (Kilgard, 2012) and preserving established sensorineural representation of those sounds.

While the effects of experience on AC c-Fos-IR were quite robust, we only found trending significance associated with estradiol exposure for AC and AC-hf, once we took our GLMM results into account. There are two possibilities as to why we did not observe a robust E2 effect, even if it were present. First, since our c-Fos-IR measure (assayed 90 min after sound playback began) decreased with experience, any modulation of plasticity that strengthens the effects of experience in AC would likely further decrease c-Fos-IR, which then runs the danger of being subject to a floor effect. Second, E2 may have a more obvious effect on AC responses at earlier stages of experience learning about the social meaning of the sounds, and our 5-day time point misses this. Future studies will need to resolve this uncertainty.

### 4.3. Correlation among brain regions

A correlation between cortical and subcortical brain regions was not observed (Fig. 5), regardless of whether the sounds were novel or familiar. This lack of correlation was not just due to errors in our ability to measure c-Fos-IR, since we did find a strong correlation between AC fields (Fig. 5A), which can be explained as a consistent, within-individual, sound-driven neural response. The lack of a strong correlation between c-Fos-IR in auditory processing regions and LC (Fig. 5B–C), despite both brain areas being sound-responsive, presumably reflects the fact that canonical sensory pathways do not directly feed into LC, which instead is integrating both external and internal factors, producing arousal (Samuels and Szabadi, 2008). Moreover, the influence of LC’s widespread projections on the cochlear nucleus (Ebert, 1996; Klepper and Herbert, 1991) and sensory neocortex (Jones and Yang, 1985; Berridge and Waterhouse, 2003), is only one potential modulator of auditoryevoked genomic responses in the AC. Any AC c-Fos-IR correlation based on this single direction of connectivity is bound to be weak. Intriguingly though, a previous study did find that pairing exogenous electrical stimulation of the LC with novel auditory stimuli in mice leads to plasticity in LC electrophysiological activity, and longlasting improvements of AC electrophysiological responses to the paired stimuli (Martins and Froemke, 2015). This raises the possibility that a stronger LC-AC correlation might be observed between an animal’s initial sensory-evoked LC c-Fos-IR when sounds are novel, and its later AC c-Fos-IR when the sound is familiar. Nevertheless, our results showing different patterns of c-Fos-IR across our experimental groups suggests at a minimum that the genomic response of these two sound processing and plasticity regions is situation-dependent.

## 5. Conclusion

We sought to determine how estradiol, social experience and the activity of a key neuromodulatory area contributes to enhancing processing of social auditory stimuli. We found further evidence for neural plasticity at key sites of auditory processing (AC-hf and A2) as social stimuli transitioned from being novel to familiar. Moreover, social sound-evoked activation of an important neuromodulatory center for sensory plasticity, LC, was not directly coupled to activity levels in cortical regions processing sounds, but instead reflected how behavioral arousal elicited by the sound can be affected by estradiol and prior sound experience. Together, these results highlight how the specific combination of internal hormonal state and past experience differentially impacts how individuals process the same sound (Fig. 6D).

## Figures and Tables

**Table 1:** GLMM confidence-interval, *z*-value and *p*-value results. AGLMMwas run using all available data in the LC, AC-hf and A2 to determine the fixed effects of estrogen, experience and possible interactions on c-Fos-IR.

Fixed Effect	z-value	*p*-value	Confidence Interval	
			Lower	Upper
E2 on AC-hf:	−0.4981	0.115	−1.125	0.035
E2 on A2:	−0.1163	0.6207	−0.574	0.343
E2 on LC	−0.5921	0.0780	−1.275	0.060
CC experience on AC-hf	−1.0584	5.50e-06***	−1.498	−0.550
CC experience on A2	−0.5172	0.0180*	−0.935	−0.055
CC experience on LC	−0.4041	0.2211	−1.046	0.224
Hormone/Experience Interaction in AC-hf	−0.298	0.6917	−0.997	0.570
Hormone/Experience Interaction in A2	−1.109	0.3042	−0.990	0.570
Hormone/Experience Interaction in LC	1.4002	0.0030**	0.447	2.344

## Abbreviations:

LC: locus coeruleus
c-Fos-IR: c-Fos immunoreactivity
AC: auditory cortex
E2: estrogen/estradiol
NE: norepinephrine
AC-hf: high frequency region within the core auditory cortex
